# The use of random forests modelling to detect yeast-mannan sensitive bacterial changes in the broiler cecum

**DOI:** 10.1038/s41598-018-31438-x

**Published:** 2018-09-05

**Authors:** A. Corrigan, N. Russell, M. Welge, L. Auvil, C. Bushell, B. A. White, R. A. Murphy

**Affiliations:** 1 Alltech Biotechnology, Sarney, Summerhill Road, Dunboyne, County Meath Ireland; 2 University of Illinois at Urbana-Champaign, Urbana, Illinois USA

## Abstract

In this study, sequencing of the 16S rRNA gene targeting the V4-V6 regions was conducted to assess the cecal microbial alterations in response to dietary supplementation with a yeast derived mannan rich fraction (MRF) in standard commercial broiler production settings across four separate broiler trials. The resulting data was analysed to identify consistent changes in the bacterial community structure of the broiler cecum in response to MRF supplementation. Subsequently, the datasets from each individual trial were pooled and analysed for differences between control and MRF supplemented diets at day 35 posthatch. The results from this analysis showed that Phylum Firmicutes was decreased and Phylum Bacteroidetes was increased across all four trials at day 35 posthatch when compared to the control. An extension of the random forest bioinformatics approach to discover a highly relevant set of microbial operational taxonomic units (OTUs) which are indicative of MRF supplementation in the broiler cecum was then used. This approach has enabled the identification of a novel set of yeast-mannan sensitive bacterial OTUs in the cecal microbiome. This information will be helpful in developing potential future nutritional strategies and will be favourable to the poultry industry.

## Introduction

The health and nutritional state of the broiler is largely interlinked with the gastrointestinal (GI) microbiome. The GI microbiome contains complex populations of microbes that affect the physiology and health of the host^1^. It has both direct and indirect effects on gut morphology, the pathogenesis of disease, immune response, nutrient digestion and uptake^2^. Microbial interactions influence the intestinal environment and as a consequence affect the development and responses of the host immune system against pathogenic and non-pathogenic bacteria^3^.

Prebiotics have been used as gut microbial modifiers to enhance the host’s natural defence by modulating the GI microbiome^4^. Prebiotics are defined as ‘selectively fermented ingredients that allow specific changes in both the composition and/or activity in the GI microbiome which in turn confer benefits upon host well being and health’^5^. As prebiotics, mannan rich fractions (MRFs) are derived from the yeast cell wall of *Saccharomyces cerevisiae*, specifically the mannoprotein portion of the outer layer. MRF’s prebiotic effect on the gut microbiome benefit the host to allow better feed digestion and absorption, which in turn aid the bird in performing more closely to genetic potential^6–8^.

Recent studies in many animal species have shown that the GI microbiome mediates key physiological processes and in doing so can be both detrimental and beneficial to the host^9,10^. However, one consistent observation when studying the GI microbiome is the significant inter-individual variation arising from both genetic and environmental influences^11,12^. This variation makes it difficult to identify key microbial species that affect the host health or that may be affected as a result of a nutritional intervention. In addition, the complexity of the microbial community and the ability to characterise and culture these same microbes adds difficulty to identifying microbial species indicative of health status. For example, the phyla Firmicutes and Bacteroidetes dominate the broiler cecum microbiome, which is estimated to contain between 500 and 1,000 species with a large percentage of these species yet to be identified^13^. Early GI microbiome studies focused on culture-dependent techniques. However, the advent of molecular techniques like high-throughput sequencing has revolutionised our ability to characterise the microbiome. Within most complex communities a small number of phylotypes dominate the population structure hiding the appearance of distinct but lowly abundant microbial taxa^14^. Therefore in order to compare the microbial populations of these complex communities and the influence of supplements, it is necessary to compare thousands of PCR amplified sequences to detect differences in the rare taxa^15^. Advances in bioinformatic techniques are now making it possible to identify differences in these distinct but low abundant microbial taxa and implicate them with a physiological outcome^16,17^.

These recent advances in biological data acquisition and sequencing technologies are enabling identification of thousands, sometimes millions, of features for a sample^18^. A common desire is to combine these data with important metadata and identify the features relevant to understanding a phenotype of interest. The focus of this research was the selection and ranking of features (OTUs) that are relevant to a phenotype of interest (MRF supplemented microbiome) from a number of metagenomic sequencing datasets. A key component underlying our approach is the use of novel analytical methods that are specialised for this type of scenario. An alternative approach from the domain of machine learning called Random Forest (RF) has been proposed for prediction, feature selection, and feature ranking in fields related to computational biomedicine^19^. An Extended Conditional Inference Forest (ECIF) approach, developed by our team to remove variables less relevant than random predictors and used successfully on multiple applications, is a novel method for better feature selection and ranking accuracy, which are critical tasks for addressing the challenges outlined above. It was based on the Boruta Package^20^. This method has been used by the team successfully for other research efforts and was applied in this analysis^21,22^.

In this study we analysed four broiler experiments to identify consistent changes in the bacterial community structure in the cecum in response to dietary supplementation with an MRF supplement. Two of these trials had previously been analysed by 454-sequencing and found that dietary MRF significantly altered the bacterial community composition (BCC) in a commercial production setting^6^. Specifically MRF supplementation altered the BCC from 7 days through to 35 days supplementation across both trials. Phylum *Bacteroidetes* appeared to be replacing Phylum *Firmicutes* as a result of supplementation, with the most noticeable effects after 35 days. PICRUSt was also used to identify differences between the functional potential of the bacterial communities as a result of MRF supplementation which indicated that alterations of the bacterial communities as a result of MRF are likely to alter the functional capability of the cecal microbiome.

Using more complex bioinformatic techniques, the extended random forests approach, we then pooled this published dataset with two additional broiler trial datasets, also sequenced using 454-technology, to identify a highly relevant set of microbial OTUs indicative of MRF supplementation. The description and understanding of nutritional strategies is central to further developing their use in poultry and for the appropriate manipulation of diets to improve poultry performance and health. This analytical approach has enabled us to discover a novel set of candidate OTUs for the classification of an MRF sensitive cecal microbiome. This work has helped understand how the GI microbiota may influence the phenotypic effects usually associated with MRF (Actigen™) supplementation in broilers. This information will be helpful in developing potential future nutritional strategies and will be favourable to the poultry industry^23^.

## Methods

### Ethics Statement

Animals were selected from commercial production units and were raised under animal welfare guidelines as set forth by the European Union. Birds were euthanized in accordance with humane killing protocols as set forth in European Union Council Regulation (EC) 1099/2009.

### Experimental Design, Sample Collection and Preservation

A total of four separate broiler trials were conducted at two different commercial production sites in Ireland (3 trials) and England (1 trial) (between late August and October). On arrival from the hatchery, 10,000 birds were randomly assigned to commercial production units where they received either a control standard commercial corn-soy diet or a standard diet plus MRF (Actigen™, Alltech Inc, Nicholasville, Kentucky) at the manufacturers recommended inclusion rates (800 g t^−1^ starter ration and 400 g t^−1^ grower ration). The birds were reared as per typical commercial production conditions receiving feed and water *ad libitum*. All other conditions were kept uniform among sheds. At days 7, 21 and 35 post-hatch the intact cecal pouches of 12 randomly selected birds per shed were euthanized and cecal contents were placed into sterile tubes containing sterilised 20% (w/v) maltodextrin which acts as a lyoprotectant. The tubes were frozen on dry ice, transported within 8 hours, lyophilised and stored at −80 °C. Animals were euthanized by cervical dislocation in accordance with humane killing protocols as set forth in EU Regulation 1099/2009.

Two of these trials (2 from Ireland) have been analysed previously for alterations in bacterial community structure as a result of yeast-mannan supplementation and the findings published^6^. For the purpose of this study, the two trials which had not been analysed previously (herein called Trial A (Ireland) and Trial B (England)) were now analysed separately for alterations in bacterial community structure as a result of yeast-mannan supplementation. Following this analysis, sequences from the day 35 timepoint for all four trials were then pooled to assess for the overall broad scale alterations in BCC. Finally, this pooled sequence dataset of four trials was then analysed using the extended random forests approach to enable the identification of bacterial OTUs which were consistently altered as a result of yeast-mannan supplementation.

### Nucleic acid extraction and PCR amplification

Total DNA was extracted in triplicate from each cecal sample using a modified cetyltrimethylammonium bromide (CTAB) extraction method as previously described^6^. This method uses the ionic detergent CTAB to disrupt cell membranes and a chloroform-isoamyl alcohol mixture that separates contaminants into the organic phase and nucleic acids into the aqueous phase. Resulting DNA was purified using a High Pure PCR product purification kit (Roche, Basel, Switzerland) according to the manufacturer’s instructions and was eluted in a final volume of 50 μL.

The V4-V6 region from the bacterial 16S rRNA operon was amplified from cecal DNA using a universal primer set, 16S-0515F (5′-TGYCAGCMGCCGCGGTA-3′) and 16S-1061R (5′-TCACGRCACGAGCTGACG-3′) tailed on each end with the Roche multiplex identifiers (MIDs). This barcode-based primer approach allowed sequencing of multiple samples in a single sequencing run without the need for physical partitioning. PCR conditions and reagents were similar to those described previously, and a standard concentration of 50 ng of cecal DNA was used in each reaction^7^. PCR products were purified using a High Pure PCR product purification kit (Roche, Basel, Switzerland).

### Sequencing, OTU picking and phylogenetic diversity analysis

Sequencing of the V4-V6 16S rRNA PCR products from the two previously unpublished trials was carried out as described by^6^. Sequencing was carried out on a Roche 454 GS-FLX titanium platform with average read lengths of approximately 530 bp generated. Following sequencing all barcodes were sorted, removed and reads were quality assessed. The MIRA v. 3.2 assembler was used to assemble the resulting forward and reverse sequences, typically with overlaps over 90% of their length, into contigs and singletons, with a 98% sequence similarity requirement. The assembler has a 454 specific error model and is able to correct for base call errors. Combining forward and reverse sequences, it recovers the high quality V4-V6 amplicon consensus sequence, even for low abundant taxa. For taxonomic assignments, BlastN analysis was used on the assembled reads against an in-house curated version of the RDP database (Michigan State University) release 10.29, containing only non-redundant sequence entries with sufficiently detailed phylogenetic assignments^24,25^. The best 25 blast hits per contig or singleton were assigned to OTUs from the NCBI taxonomy with MEGAN v.4. MEGAN considers both blast score and taxonomic level of the blast hits in order to pick the appropriate OTU^26^. OTU counts were corrected for sequence numbers per contig, so as to obtain final OTU tables^26^. The Generalized Unifrac approach (R Bioconductor v. 2) was used to estimate pairwise distances between samples and establish beta diversity after alpha rarefaction^27^. PCA analysis was performed to establish two-dimensional projections of samples, reflecting time point and control/treatment status.

### Construction of datasets for Random Forest classification

The challenge of finding relevant OTUs was achieved through a pipeline of binary classification and feature selection^28^. The primary algorithm used was the Extended Random Forest (ERF). The ERF algorithm builds a random forest decision model for a classification problem and through the usage of permutation tests, identifies which OTUs have the most predictive value. The ERF approach has the benefit of capturing variable importance even when there is an order of magnitude more variables than samples, as well as considering variable importance in the presence of multivariate interactions with other variables^29^.

To understand what OTUs were significant in differentiating MRF supplemented from control groups, a binary classification was established between the two groups and the ERF algorithm used. This step was followed by stability analysis and feature permutation to identify a minimal optimal set of highly relevant OTUs. The original feature set was extended with a permuted set of features, called shadow features. For each original feature, a shadow feature was created by randomly permuting the values from all observations. The measure for feature relevance was the likelihood that a feature has a higher mean variable importance metric (VIM) than its shadow feature. This was estimated by the p-value of a one-sided t-test between each feature and the shadow with the highest mean VIM.

### Phylogenetic tree construction

OTUs that were considered to be highly relevant by ERF were used to construct phylogenetic trees using tools in the Ribosome Database Project^25^.

## Results

A recent study by^6^ identified that MRF supplementation consistently altered the BCC across two concurrent trials^6^. The goal of this research was to gain new insights into the specific and reproducible effects of MRF supplementation on the cecal BCC. We sought to merge the existing knowledge of these two published trials with two additional broiler trials to identify the dominant changes in the principal taxonomic categories and also to assess if we could identify a consistent set of MRF sensitive bacterial OTUs in the broiler cecum using an extended RFM of classification.

### Dietary supplementation with MRF alters bacterial community composition of the broiler cecum

**Figure 1:**
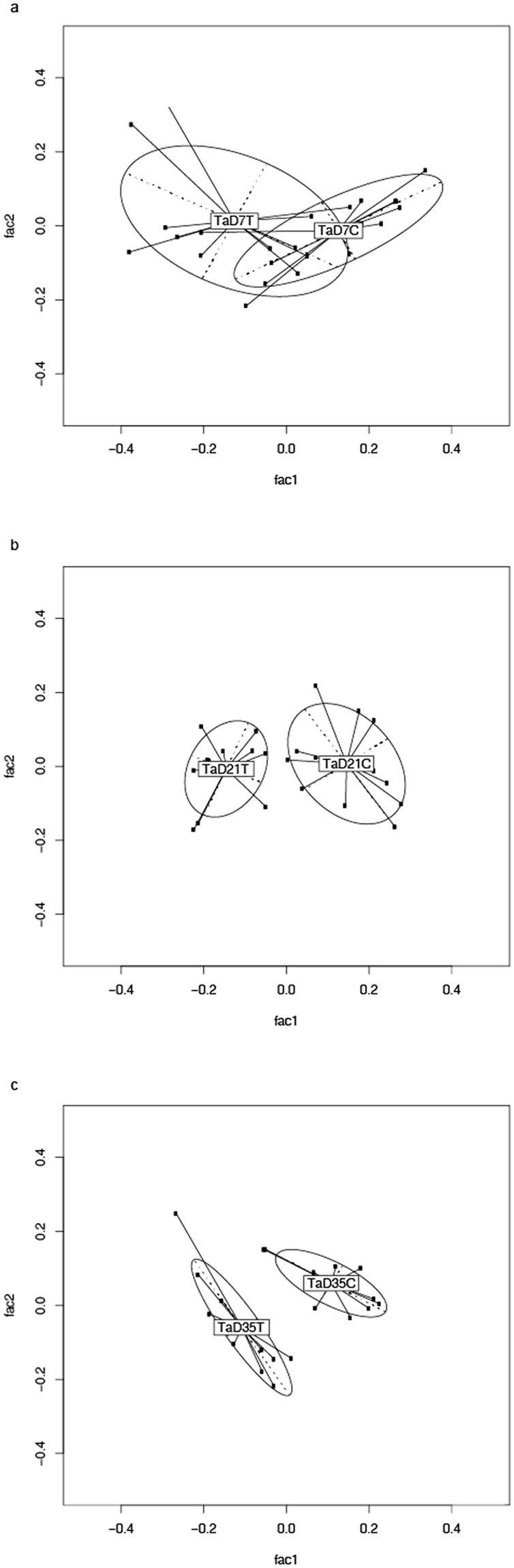
(**a**–**c**) PCA analysis was performed on pairwise distance estimates obtained from Megan 4 OTU picking followed by Generalized Unifrac analysis to assess for differences between control and MRF supplemented groups at each time point from Trial A. (a = 7 days posthatch, b = 21 days posthatch, c = 35 days posthatch, n = 12 for each group). T = trial, D = day, C = control, T = MRF supplemented.

**Figure 2:**
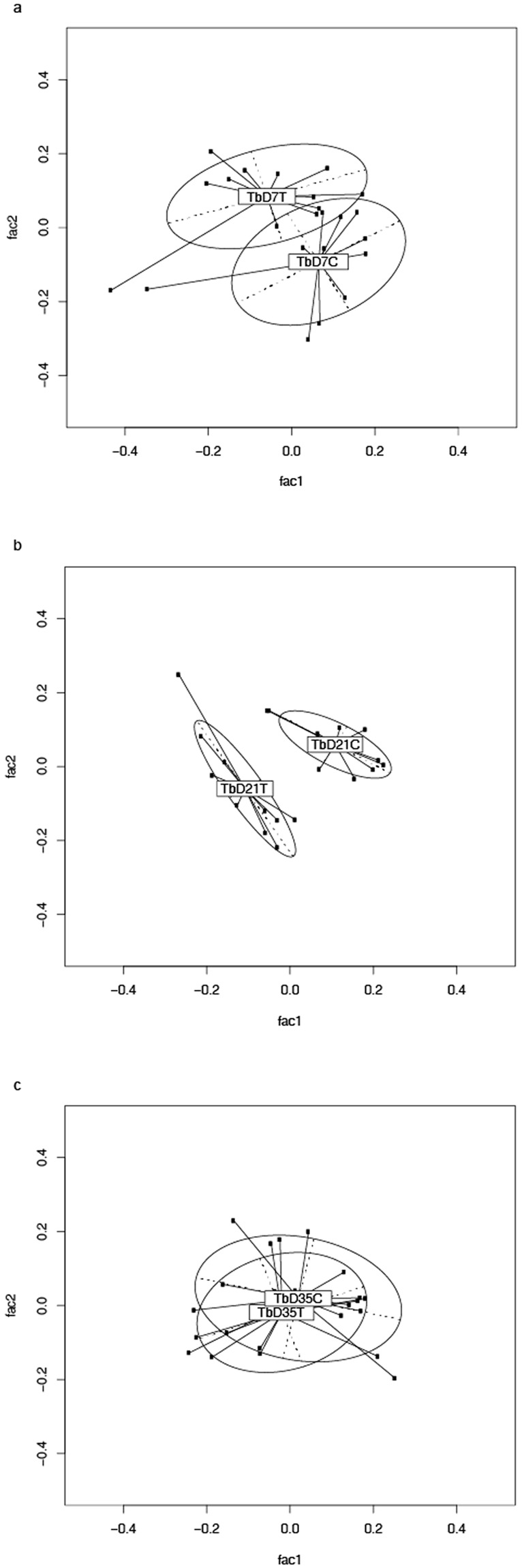
(**a**–**c**) PCA analysis was performed on pairwise distance estimates obtained from Megan 4 OTU picking followed by Generalized Unifrac analysis to assess for differences between control and MRF supplemented groups at each time point from Trial B. (a = 7 days posthatch, b = 21 days posthatch, c = 35 days posthatch, n = 12 for each group). T = trial, D = day, C = control, T = MRF supplemented.

The influence of MRF supplementation on the BCC of the broiler ceca from two previously unanalysed trials, one based in Ireland (trial A) and one based in England (trial B), was analysed by 454 sequencing. Approximately 500,000 high quality reads were obtained from 144 samples collected from the cecal contents of 12 birds on days 7, 21 and 35 posthatch for each of the control and supplemented groups. The sequences were analysed as described by^6^ and taxonomically assigned at the 3% distance level^6^. Alterations in the BCC identified in each trial and at each time point were visualised using GUniFrac from the R package. The permutational multivariate analysis of variance (PMAoV) was used to test for significance. Results indicated that cecal BCC was significantly altered as a result of dietary MRF in both trials. MRF supplementation significantly altered BCC at days 7, 21 and 35 posthatch in trial A (Fig. 1a–c) whilst significant alterations at days 7 and 21 but not at 35 days posthatch were observed for trial B (Fig. 2a–c) (P < 0.05).

### Dietary MRF consistently alters bacterial Phyla

**Figure 3:**
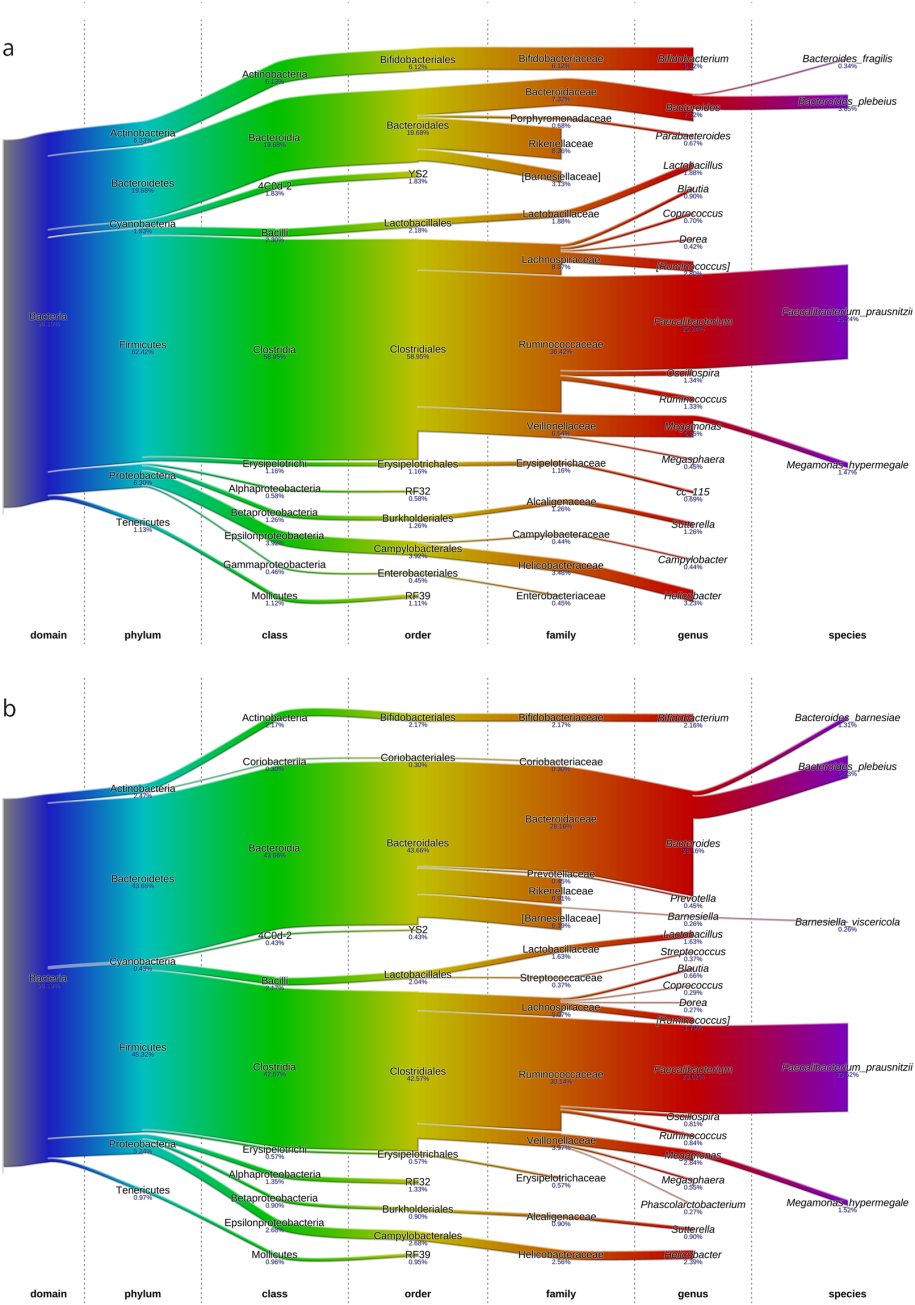
Bacterial phyla distributions (percentage relative abundance) at day 35 posthatch in control (**a**) and MRF supplemented (**b**) groups for the four broiler trials combined (n = 48 for each group).

Following confirmation that MRF supplementation significantly altered BCC in trials A and B it was decided to pool these datasets with the previously published sequences^6^ in order to assess trends in alterations of the cecal microbiome at the taxonomic level. This involved combining and rebinning all sequence datasets to assess overall effects on BCC. Analysis was limited to day 35 posthatch given the results from previous experiments which showed the effect of MRF supplementation was strongest at this timepoint. BlastN analysis against the RDP database was used for taxonomic assignments. Eleven bacterial phyla were identified from this combined data set. Phlya identified included Firmicutes, Bacteroidetes, Proteobacteria, Actinobacteria, Cyanobacteria Tenericutes, Euryarchaeota, Deferribacteres, Verrucomicrobia, Lentisphaerae, and Thermotogae in order of relative abundance. The top six most abundant phyla identified accounted for approximately 98% of all sequences in both the control (Fig. 3a) and MRF-supplemented groups (Fig. 3b). Comparison of the microbiota composition of the combined datasets revealed stark alterations in composition as high as at the phylum level. The relative abundance of Phylum Bacteroidetes increased in the MRF-supplemented birds by an average of 24%; Phylum Firmicutes was decreased on average by 17%, and the Phyla Actinobacteria, Proteobacteria, Cyanobacteria and Tenericutes were all decreased by as a result of MRF supplementation relative to the controls. At a family level, the main increases were observed in the Coriobacteriaceae, Bacteroidaceae, Prevotellaceae, Barnsiellaceae, and Streptococaceae whilst decreases in families Bifidobacteriaceae, Rikenellaceae, Porphyromonadaceae, Lactobacillaceae, Ruminococcaceae, Veillonellaceae, Erysipelotrichaceae, Alcaligenaceae, Campylobacteraceae, Helicobacteraceae, and Enterobacteraceae were observed in the MRF supplemented group compared to the control.

### Identification of yeast-mannan sensitive bacterial OTUs in the cecal microbiome

**Figure 4:**
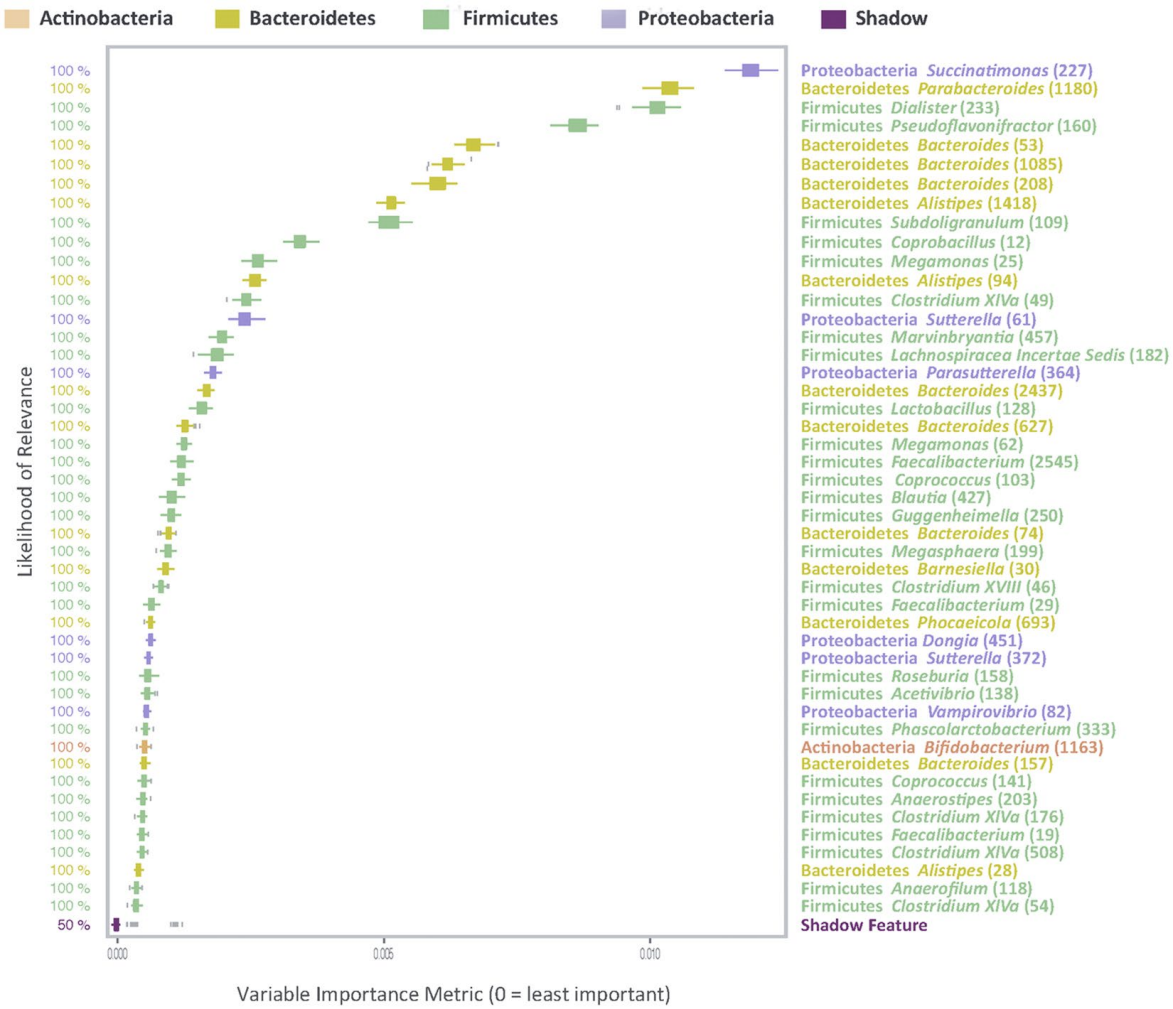
Extended conditional inference forest variable importance matrix. OTUs and phylogenetic associations identified as important at defining differences in control and MRF supplemented cecal microbiome.

**Figure 5:**
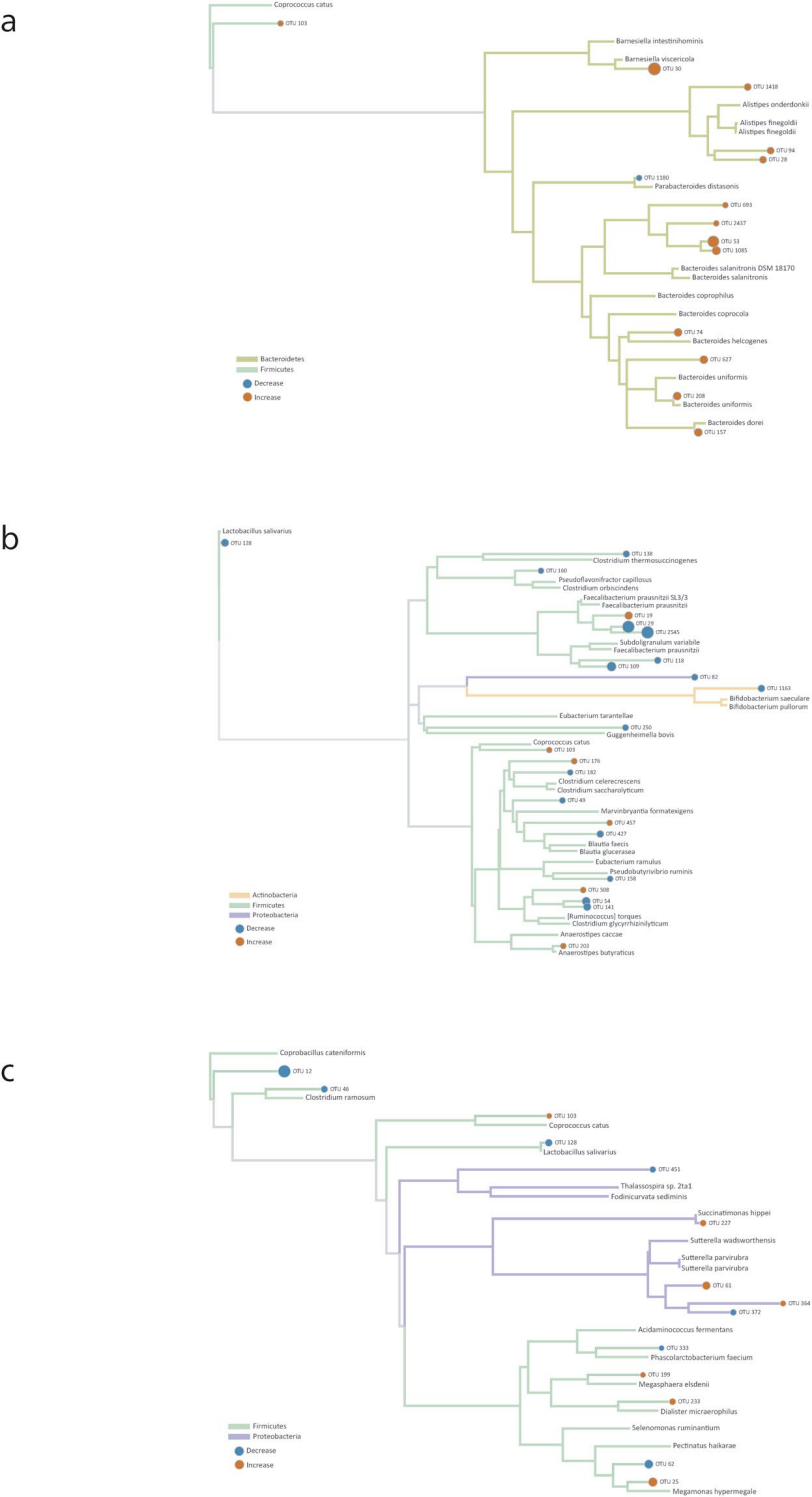
(**a**–**c**) Phylogenetic trees of OTUs from all 4 datasets identified as significant when comparing Control and MRF supplemented BCC. The orange circles represent OTUs that were more abundant in the MRF supplemented BCC, and blue circles represent OTUs that were less abundant in the MRF supplemented BCC. The size of the circles is directly proportional to the magnitude of the abundance. All of these OTUs were considered to be significant features by ERF. Panel a shows the order Bacteroidales OTUs with *Coprococcus catus* as the outgroup. Panel b shows the orders Clostridiales, Bifidobacteriales, and Bdellovibrionales with *Lactobacillus salivarius* as the outgroup. Panel c shows the orders Burkholderiales, Aeromonadales, Selenomonadales, Erysipelotrichales, Lactobacillales, and Kiloniellales with *Coprobacillus cateniformis* as the outgroup.

Results from 454 sequencing showed that the BCC was altered in four broiler trials and two locations. In addition, at 35 days posthatch these alterations were mainly due to increases in phylum Bacteroidetes and decreases in phylum Firmicutes. To identify bacterial OTUs that were indicative of differentiating MRF supplemented and control BCC we employed a supervised machine learning approach; the conditional extended random forest approach. This method also applies an importance score to each OTU identified, indicating how important the selected OTUs are at contributing to differences. In our analysis we considered an OTU to be highly predictive if it’s importance score was at least 0.001. This method identified 47 individual bacterial OTUs across all four trials as being important at defining differences in control and MRF supplemented cecal microbiome with a likelihood of 100% (Fig. 4). Many other OTUs were identified as important (S1 Table) for defining differences in control and MRF supplemented microbiome composition with likelihoods of >97%. It must be noted that the cut-off point at 100% is an arbitrary figure as those ranking higher than the shadow feature are still relevant. We then assessed whether OTUs identified as relevant in distinguishing BCC were either over- or under-represented in MRF supplemented groups. We found 23 OTUs were decreased and 24 OTUs were increased in MRF supplemented birds relative to the control. OTUs were then assessed phylogenetically to identify specific trends related to MRF supplementation (Fig. 5a–c). The 47 OTUs identified were members of 4 bacterial phyla; Bacteroidetes (13), Firmicutes (27), Proteobacteria (6) and Actinobacteria (1).

OTUs of the phylum Bacteroidetes showed a general trend that identified all but one of 13 OTUs increased in response to MRF supplementation (Fig. 5a). The majority of these OTUs (8 of 13) clustered within the family Bacteroidaceae, and were related to genus Bacteroides. Of the remaining Bacteroidetes-related OTUs which were increased in response to MRF supplementation, three were phylogenetically clustered within family Rikenellaceae, genus Alistipes and one OTU clustered with family Porphyomonadaceae, genus Barnsiella. One OTU relating closely to *Parabacteroides* spp, was shown to be decreased within this phylum (family Porphyomonadaceae).

Within the phylum Firmicutes, 27 OTUs were identified as important at distinguishing between MRF supplemented and control diets. The majority of the OTUs (18) were decreased by dietary MRF while nine were increased (Fig. 5 Panel b,c). OTUs identified were phylogenetically clustered into three main families; Lachnospiraceae, Ruminococcaceae and Veillonellaceae, while two OTUs clustered into family Erysipelotrichaceae and a single OTU each clustered into families Lactobacillaceae and Clostridiaceae. Within family Lachnospiraceae, a diverse family within the phylum Firmicutes, OTUs were differentially affected in response to MRF, with six OTUs decreased and five increased relative to the control (Fig. 5b). Two OTUs clustered strongly with known bacterial genera closely related to *Coprococcus catus* and *Anaerostipes butyraticus* with both increasing in birds given dietary MRF.

The Ruminococcaceae another diverse family within the phylum Firmicutes and together with the Lachnospiraceae make up two of the most abundant families from the order Clostridiales within the intestine^30^. OTUs identified within this family showed a discernible pattern with 6 of 7 OTUs decreased in response to dietary MRF and clustered strongly with the genus Faecalibacterium (Fig. 5b).

Within family Veillonellaceae five OTUs were identified as important in response to MRF supplementation with two decreasing and three increasing (Fig. 5c). Two OTUs that increased clustered tightly with identifiable bacterial genera *Megasphaera elsdenii* and *Megamonas hypermegale*.

A decrease in one OTU of the family Lactobacillaceae, whose closest match was *Lactobacillus salivarius* and a decrease in two OTUs of family Erysipelotrichaceae which matched closely to *Coprobacillus* spp, and *Clostridium ramosum* was observed in response to MRF supplementation. A decrease in one OTU of the family Clostridiaceae, whose closest match was *Guggenheimella bovis* was observed in response to MRF supplementation (Fig. 5b,c).

There were six OTUs of the phylum Proteobacteria identified as important at distinguishing dietary supplemented microbiota composition. Three of these OTUs increased while three decreased, however, there was no clear pattern in response to MRF supplementation (Fig. 5b,c). The OTUs clustered within the bacterial families Succinovibrionaceae, Alcaligenaceae, Rhodospirillaceae and one poorly defined OTU.

One OTU of the phylum Actinobacteria was identified as important at distinguishing between supplemented groups (Fig. 5b). This OTU was decreased in response to MRF supplementation and clustered within family Bifidobacteriaceae with the closest match to genus *Bifidobacterium* spp.

## Discussion

In studies of the GI microbiome’s effect on health and productivity of the broiler much of the research to date has focused on optimising microbial community composition to aid the bird’s natural defences against pathogens such as *Salmonella* spp. and pathogenic Clostridia^2,31–34^. These infections cause poor flock health, reduced performance and increased mortalities resulting in economic losses to producers^3,35^. In addition, many of the studies into the effects of prebiotics on the GI microbiome and on host health have focused on specific beneficial bacterial populations such as *Lactobacillus* spp., or *Bifidobacterium* spp., which are known to affect intestinal health^36,37^. However, while the effects of prebiotics on poultry health have been well reviewed in both of these regards, there has been less research concerning their impact on the microbiome as a whole, on host health and performance. In recent years, some studies have shown that MRF supplementation in poultry alters the overall bacterial community structure^4,38^. The results demonstrated in Figs 1 and 2 have substantiated these studies and further confirmed that community structure changes as a result of MRF supplementation regardless of location, age and production environment^6–8^. The authors interpretation of these results suggests MRFs can be effective for manipulation of the cecal microbiome in the starter, grower and finisher diets as alterations were observed from as early as 7 days through 35 days of supplementation^6–8^. Understanding whether changes in BCC in response to diet are consistent and if they might contribute to host health is important as there is mounting evidence that the GI microbiome influences animal performance^39^. A previous broiler study showed there is a correlation between shifts in cecal microbiome composition and the efficiency of feed nutrient conversion^40^. As such it may prove beneficial to spread the scope of future studies beyond focusing on the ‘traditional’ bacterial genera to better understand nutritional effects on other gut microbes that could contribute to broiler performance^2,39^. If optimization of microbial balance in the cecal environment leads to good performance, it will be of great importance to establish or develop strategies to provide a competitive advantage for representatives of taxa identified as beneficial. It is with this goal in mind that we sought to taxonomically identify what bacteria were contributing to this shift in composition as a result of MRF supplementation across all four studies.

The sequencing datasets of all 4 studies were pooled and taxonomic analysis showed notable shifts at the phylum level between control and MRF supplemented microbiomes. A review by^41^ found that phylum Firmicutes dominated the broiler cecal microbiome accounting for an average of 70% of all sequences with Bacteroidetes (12%) and Proteobacteria (9%) accounting for the second and third most abundant phyla. These relative abundances are similar to that observed in the control groups of the four broiler trials discussed in this study. However, in the cecal microbiome of the MRF supplemented birds across the four trials assessed in this present study, a large shift in the bacterial taxonomic composition to a Bacteroidetes prominent profile was observed. Changes in bacterial profile corresponding to an increase in bacterial complexity have generally been associated with improved host health and microbiome stability with decreased bacterial complexity associated with higher incidence of disease^42–45^. In particular, increases in the phylum Bacteroidetes within the gut microbiome have been associated with many favourable outcomes. For example, intestinal microbial health in humans has been restored through microbiota transplantation from a healthy to a diseased individual resulting in a recovery of the microbial balance in the gut^46^. The recipient changed from a Firmicutes and Bacteroidetes deficient configuration to a community dominated by *Bacteroides* spp, resulting in the disappearance of disease like symptoms in the patient^46^. If a similar microbial balance in the poultry microbiome could lead to reduced incidence of enteric disease, thereby reducing the need for antibiotic use, this would be beneficial to both producers and consumers^2,47^. A study by^48^ examined the BCC changes in high feed conversion ratio (FCR) and low-FCR broilers and found that those birds with a lower FCR tended to be associated with higher levels of *Bacteroidetes*^12,48^. Enriched Bacteroidetes and depleted Firmicutes phyla were also noted in differences in intestinal microbiota between European and African children with the latter having higher levels of Bacteroidetes^49^. This has been hypothesised to be linked with differences in diet with increased Bacteroidetes allowing improvement of energy uptake from a typical fibre-rich African diet. Increased fibre digestion as a result of an increase in Bacteroidetes related bacteria could be beneficial to poultry farmers as more energy could be extracted from the diet resulting in improved feed efficiency^50^. A more varied taxonomic composition may also influence the stability of the microbiota due to an increased functional capacity that accompanies a more complex microbiome and allowing for quicker adaptation to change^51^. Previously, shifts in Firmicutes-dominant to Bacteroidetes-dominant bacterial profiles have been predicted to alter the functional potential of the microbiome by enriching pathways predicated to be associated with energy metabolism and carbon fixation in broilers^6^. There is much evidence in the literature to suggest that MRF supplementation is linked with improved broiler health and performance^52,53^ however, whether a link exists between this consistent phylogenetic shift as a result of MRF supplementation and host health and performance is yet to be elucidated.

Having observed this consistent higher level taxonomic shift between control and MRF supplemented birds we decided to seek methods of analysis which would allow identification of a specific set of bacterial OTUs which distinguished the MRF supplemented microbiome from the control. An extension of the random forest approach was therefore chosen to identify bacterial OTUs consistent with MRF supplementation from the sequencing datasets. This bioinformatic method was developed to analyse high dimensional omics datasets to allow identification of biomarkers. Using this classifier we have identified a consistent set of bacterial OTUs that are sensitive to MRF supplementation across all four trials. OTUs were identified from 4 main bacterial phyla and represent candidate biomarkers of MRF supplementation, with many having noted benefits in the literature for improved broiler health and performance. For example OTU candidates identified from phylum Bacteroidetes were mostly associated with family Bacteroidaceae and related mainly to *Bacteroides* spp. All eight of these OTUs increased in response to MRF supplementation. *Bacteroides* spp., are common bacteria in the intestine, involved in many important metabolic activities, including polysaccharide degradation, carbohydrate fermentation, and prevention of pathogen colonization^54,55^. *Bacteroides* spp., along with *Alistipes* spp., of the family Rikenellaceae are some of the main short chain fatty acid producers in the intestine^56^. An increase in short chain fatty acid production could be beneficial for gut health^57,58^. *Barnesiella* spp., have been shown to inhibit colonization by vancomycin-resistant Enterococci in mice^59^. The single Bacteroidetes-related OTU that decreased in response to dietary MRF clustered with the genus Parabacteroides of the family Porphyromonadaceae, a common bacterium identified in the poultry cecum for which the function is unknown^41^. In general, the results shown here suggest that phylum Bacteroidetes are positively influenced by MRF supplementation in the broiler cecum.

The influence of MRF supplementation on candidate OTUs identified from the phylum Firmicutes is not as clear as that of the Bacteroides phylum. Within this very diverse phylum OTUs were clustered into three main bacterial families; Lachnospiraceae, Ruminococcaceae and Veillonellaceae. Within the family lachnospiraceae it was difficult to define a clear trend with regard to MRF supplementation as 6 OTUs decreased and 5 OTUs increased. Two OTUs clustered strongly with the known genera *Coprococcus catus* and *Anaerostipes butyraticus*. Increases in OTUs related to these species may have a positive influence on broiler production by providing energy to the host in the form of short chain fatty acids. *Coprococcus catus* produces butyrate from fructose and produces propionate from lactate. *Anaerostipes butyraticus* is a known butyrate producer which is important for gut health^60^. These OTUs may be part of a bacterial network responsible for biosynthesis of the major microbial metabolites resulting from carbohydrate fermentation.

Candidate OTUs identified from the family Ruminococcaceae appeared to show a more specific trend as a result of dietary MRF inclusion with 6/7 OTUs decreased. These OTUs tended to cluster strongly with the genus *Faecalibacterium* spp. which are frequently found in the mammalian gut environment and have commonly been associated with gut health^30^. In studies of the poultry microbiome, correlations have been found between the co-occurrence of *Campylobacter spp*. and *Faecalibacterium spp*.^61–63^. These authors have shown that *Faecalibacterium* spp., presence typically indicates increased *Campylobacter* spp. presence. This might suggest a positive role for decreased Faecalibacterium-related OTUs in the broiler cecum, as a reduction in the presence of *Campylobacter* spp. in the broiler intestine is of significant interest to poultry producers. Further in-depth studies would be required to confirm these associations.

The response of candidate OTUs from family Veillonellaceae to MRF supplementation was also variable with two OTUs decreased and 3 OTUs increased. Of possible interest are two OTUs which clustered strongly with *Megasphaera esldenii* and *Megamonas* spp. *Megasphaera esldenii* has been investigated as a probiotic for ruminants as it may provide benefits for energy balance and animal productivity owing to its capability of producing various volatile fatty acids in the rumen^64^. *Megamonas* spp. have been associated with *Campylobacter jejuni* suppression in broilers, increasing bacterial OTUs which suppress this zoonotic pathogen would be beneficial to both poultry producers and human health^65^.

A decrease in one OTU of the family Lactobacillaceae, whose closest match was *Lactobacillus salivarius* was observed in response to MRF supplementation. *Lactobacillus* spp. are noted as probiotic bacteria and typically an increase in this OTU would be desirable^66^. However, although there are some strains of Lactobacillus known to improve performance some are retailed as weight loss probiotics and others reported to reduce obesity^67^. In a study by^68^ they found that some *Lactobacillus* spp, correlated with a negative effect on performance in poultry. This highlights that specific OTUs need to be identified and verified as having either beneficial or negative effects on health and performance in each candidate host and that not all strains or species of a particular genus can have the same effect^67^. A decrease in one OTU of the family Clostridiaceae, whose closest match was *Guggenheimella bovis* was observed in response to MRF supplementation. This bacterium has been associated with digital dermatitis in cows leading to production losses for farmers^69^. This genus of bacteria is not frequently identified in the poultry GI tract. Two OTUs related to family Erysipelotrichaeae were also shown to decrease in response to MRF supplementation relating to *Clostridium ramosum* and *Coprobacillus cateniformis*. Members of the family Erysipelotrichaeae have been implicated in weight gain in obese humans and also identified as potential probiotic candidates in poultry due to their association with improved FCR^70,71^. However, this is a diverse family with varying functional capabilities making it difficult to suggest a potential positive or negative role for its reduction in terms of MRF supplementation.

There were six OTUs of the phylum Proteobacteria identified as important at distinguishing dietary supplemented microbiota composition. Some of these OTUs increased while others decreased however, there was no clear pattern in response to MRF supplementation (Fig. 5b,c). The OTUs clustered within the bacterial families Succinovibrionaceae, Alcaligenaceae, Rhodospirillaceae and one poorly defined OTU. Very little is known with regard to the possible relationship of these OTUs to poultry intestinal health. This further highlights the need to expand our scope of investigation beyond the ‘traditional’ microbiome. One OTU related to *Bifidobacterium* spp., of the phylum Actinobacteria was also shown to decrease. Similar to *Lactobacillus* spp., *Bifidobacterium* spp., are also commonly used as probiotics in poultry but not all species of *Bifidobacterium* are beneficial to improving broiler performance^72,73^. Further investigations of this specific OTU and its possible implications on host health would be required to understand the possible functional impact of its reduction as a result of MRF supplementation.

## Conclusions

Based on this large scale survey it is clear that MRF supplementation has a consistent and reproducible effect at altering BCC most notably by causing a large shift in the relative abundance of the Firmicutes and Bacteroidetes bacterial phyla. These higher level bacterial community changes have been implicated with improved host health in many species and in this case may be indicative of an MRF supplemented phenotype. More specifically, consistent differences were noted at the OTU level, mainly on OTUs related to Ruminococcaceae and Bacteroidaceae. These results were revealed using the random forest approach and would not have been detected by conventional bacterial community analysis methods due to the inherent problems with inferring associations using relative abundances^74^. The identification of diet sensitive bacterial OTUs opens possibilities to further investigate if changes in specific OTUs are implicated in specific phenotypic outcomes associated with diet. The 16S rRNA gene sequences generated in this study have established the basis for developing quantitative assays for the enumeration and identification of specific yeast-mannan sensitive OTUs.

## Electronic supplementary material

Supplementary Material

## Data Availability

Sequencing data are accessible in the European Nucleotide Archive (Short Read Archive) under accession numbers ERP009698 and ERP016138.
