# Supplementary material for: The use of random forests modelling to detect yeast-mannan sensitive bacterial changes in the broiler cecum
Source: Sci Rep. 2018 Sep 5;8:13270. doi: 10.1038/s41598-018-31438-x (PMC6125325; doi:10.1038/s41598-018-31438-x)
Supplement: Supplementary file 1 — Supplementary Material [file 41598_2018_31438_MOESM1_ESM.pdf]

The use of random forests modelling to detect yeast-mannan sensitive bacterial changes in the broiler cecum

Authors: A. Corrigan[a],*, N. Russell[b], M. Welge[b], L. Auvil[b], C. Bushell[b], B.A. White[b],. and R. A. Murphy[a]

Affiliations:

Alltech Biotechnology, Sarney, Summerhill Road, Dunboyne, County Meath, Ireland[a]

University of Illinois at Urbana-Champaign, Urbana, Illinois, USA[b]

* Corresponding author mailing address: Alltech Bioscience Centre, Summerhill Road, Sarney,Dunboyne, Co. Meath, Ireland. Phone +353 18026258. E-mail: acorrigan@alltech.com.

Running title: Yeast-mannan sensitive bacteria of the broiler cecum

ORCID: 0000-0002-7169-9061

S1 Table. OTU's identified above the shadow feature using the extended conditional inference forest method. This table shows the variable importance measure (VIM) as well as the likelihood of relevance of each OTU which was identified up to the shadow feature.

VIM	Likelihood of relevance	Phylum	Family	Genus
0.000343494	0.999999999	Bacteroidetes	Rikenellaceae	Alistipes
0.000335174	0.999999998	Firmicutes	Ruminococcaceae	Clostridium
0.000328377	0.999999995	Firmicutes	Clostridiales_Incertae_Sedis_XII	Guggenheimella
0.000313757	0.999999963	Bacteroidetes	Bacteroidaceae	Bacteroides
0.000299685	0.999999789	Actinobacteria	Bifidobacteriaceae	Bifidobacterium
0.000294663	0.999999608	Firmicutes	Lachnospiraceae	Clostridium_XlVb
0.000282161	0.999998111	Bacteroidetes	Bacteroidaceae	Bacteroides
0.000258736	0.999976094	Firmicutes	Ruminococcaceae	Acetanaerobacterium
0.000253384	0.999957538	Firmicutes	Lachnospiraceae	Lachnospiracea
0.000252876	0.99995748	Deferribacteres	Deferribacteraceae	Mucispirillum
0.000248819	0.999924494	Proteobacteria	Enterobacteriaceae	Escherichia_Shigella
0.000247063	0.999915195	Firmicutes	Lachnospiraceae	Clostridium_XlVa
0.000233651	0.999678686	Firmicutes	Erysipelotrichaceae	Clostridium_XVIII
0.000218064	0.998707216	Firmicutes	Ruminococcaceae	Ruminococcus
0.000216275	0.998414786	Firmicutes	Lachnospiraceae	Clostridium_XlVa
0.000213834	0.998180074	Bacteroidetes	Porphyromonadaceae	Barnesiella
0.000186741	0.983589984	Firmicutes	Lachnospiraceae	Clostridium_XlVa
0.000186224	0.984458482	Firmicutes	Lachnospiraceae	Clostridium_XlVa
0.000182371	0.979753633	Firmicutes	Lachnospiraceae	Clostridium_XlVa
0.000178147	0.973394826	Firmicutes	Lachnospiraceae	Blautia
0.000173971	0.965418098	Bacteroidetes	Bacteroidaceae	Bacteroides
0.000171158	0.958750826	Proteobacteria	Helicobacteraceae	Helicobacter
0.0001706	0.957539949	Bacteroidetes	Bacteroidaceae	Bacteroides
0.000162316	0.931697318	Firmicutes	Lachnospiraceae	Anaerostipes
0.000159465	0.920551806	Firmicutes	Lachnospiraceae	Blautia
0.000157489	0.912400891	Actinobacteria	Bifidobacteriaceae	Bifidobacterium
0.000157064	0.910289689	Firmicutes	Ruminococcaceae	Papillibacter
0.00015196	0.884170282	Firmicutes	Ruminococcaceae	Ruminococcus
0.000151047	0.879953132	Bacteroidetes	Cryomorphaceae	Wandonia
0.000147214	0.857059801	Firmicutes	Ruminococcaceae	Acetivibrio
0.000146962	0.85399343	Firmicutes	Lactobacillaceae	Lactobacillus
0.000146412	0.850500123	Firmicutes	Lachnospiraceae	Blautia
0.000145156	0.842860682	Proteobacteria	Bdellovibrionaceae	Vampirovibrio
0.000137956	0.787751478	Firmicutes	Ruminococcaceae	Oscillibacter
0.000125007	0.668258268	Firmicutes	Ruminococcaceae	Acetivibrio
0.000121426	0.630421695	Bacteroidetes	Bacteroidaceae	Bacteroides
0.00011948	0.609117886	Firmicutes	Lachnospiraceae	Clostridium_XlVa
0.000116933	0.580940099	Firmicutes	Lachnospiraceae	Clostridium_XlVa
0.000114307	0.551617457	Euryarchaeota	Methanomicrobiaceae	Methanosphaerula
0.000110928	0.513230105	Firmicutes	Enterococcaceae	Enterococcus
